# The Applications of Focused Ultrasound (FUS) in Alzheimer’s Disease Treatment: A Systematic Review on Both Animal and Human Studies

**DOI:** 10.14336/AD.2021.0510

**Published:** 2021-12-01

**Authors:** Xiaodan Liu, S. Sta Maria Naomi, Wu Lin Sharon, E. Jacobs Russell

**Affiliations:** Zilkha Neurogenetic Institute, University of Southern California, Los Angeles, CA, USA

**Keywords:** Focused ultrasound (FUS), Alzheimer’s disease (AD), systematic review

## Abstract

Alzheimer’s disease (AD) affects the basic ability to function and has imposed an immense burden on the community and health care system. Focused ultrasound (FUS) has recently been proposed as a novel noninvasive therapeutic approach for AD. However, systematic reviews on the FUS application in AD treatment have not been forthcoming. We followed the Preferred Reporting Items for Systematic Reviews and Meta-Analyses (PRISMA) criteria to summarize the techniques associated with safety and efficacy, as well as possible underlying mechanisms of FUS effects on AD in animal and human studies. Animal studies demonstrated FUS with microbubbles (FUS-MB) induced blood-brain-barrier (BBB) opening that could facilitate various therapeutic agents entering the brain. Repeated FUS-MB and FUS stimulation can relieve AD pathology and improve cognitive and memory function. Human studies showed repeated FUS-MB are well tolerated with few adverse events and FUS stimulation could enhance local perfusion and neural function, which correlated with cognitive improvement. We conclude that FUS is a feasible and safe therapeutic and drug delivery strategy for AD. However, FUS treatment on humans is still in the early stages and requires further optimization and standardization.

Alzheimer’s disease (AD) is one of the most common forms of dementia in the elderly. AD is characterized by extracellular amyloid plaques composed of amyloid β (Aβ) aggregates, intracellular neurofibrillary tangles (NFT) with hyperphosphorylated tau, deficits in neurotransmitters, and synaptic and neuronal degeneration. AD patients often present a series of symptoms, including decline in reasoning, loss of memory and general deterioration of cognitive capacities. Eventually, patients will lose the basic ability to deal with daily life and require around-the-clock care. According to a report from the Alzheimer’s Association, an estimated 5.8 million Americans age 65 and older are living with AD in 2020, age 85 and older account for 32% of AD patients [1]. In 2019, more than 16 million family members and other unpaid caregivers provided an estimated 18.6 billion hours of care to AD patients. Medical payments for service are more than 23 times greater to persons ≥65 years old with AD than to those without AD, thus imposing an immense burden on the community and health care system[1]. Furthermore, death from AD had a steep increase between 2000 and 2018 (increased 146.2%), making AD the fifth leading cause of death among Americans age 65 and older [1].

Despite the progress made in recent years toward understanding AD, there are no effective treatments, and no cures are available. Currently, the clinical therapeutic interest concentrates on pathological hallmarks of AD, such as Aβ and tau. Several innovative large molecule therapeutics (antibodies, proteins, gene therapeutics and stem cells) that target biomarkers of AD are under development or in clinical trials. However, the limited penetrability of the blood-brain-barrier (BBB) prevents these drugs from reaching therapeutic levels in the brain. Administering higher doses of these drugs could deliver therapeutic levels in the brain and may also increase the risk of systemic adverse effects and incur higher costs for the patient. There are several traditional methods for increasing drug delivery into the brain by either disrupting or bypassing the BBB, such as administration of hyperosmotic solutions [2], localized temperature elevation [3], localized injection of drugs and biologic agents (virus, vasoactive molecules and compounds that use innate cell-mediated transport) [4]. However, these methods are limited by poor spatial specificity, invasive methodology and require complex biochemical design, which restricts their widespread use in the clinic.

Focused ultrasound (FUS) coupled with the infusion of microbubbles (MB) (FUS-MB) has been studied in recent years and is regarded as a noninvasive approach to disrupt the BBB in a transient and reversible manner. FUS-MB could facilitate targeted accumulation of large therapeutic agents in the brain for a desired therapeutic effect [5-14]. FUS-MB induced BBB opening alone could lower the Aβ and tau burden, induce neurogenesis and neural plasticity and enhance cholinergic function, resulting in cognitive improvement in preclinical models of AD [15-22]. In addition, FUS stimulation without MB has been shown to induce neurogenesis, neuromodulation and immunogenetic response, which correlates with improvements in cognitive function and memory in preclinical models of AD [23-27]. Nicodemus et al. demonstrated no adverse events and improved both cognitive and motor scores with FUS stimulation in AD and Parkinson’s disease (PD) patients [28].

Accumulating evidence in AD animal models indicates FUS-MB drug delivery, FUS-MB treatment and FUS stimulation are safe and effective. However, there are no uniform standards for FUS parameters or hardware and a general lack of understanding of the underlying mechanisms of FUS induced therapeutic effects on AD. Nevertheless, the application of FUS on AD patients is currently undergoing phase I and II clinical trials. Promising results indicate that repeated FUS-MB treatment and FUS stimulation are well tolerated with few adverse events, thus could feasibly be applied in mild to moderate AD patients. Clinical trials involving FUS stimulation showed beneficial effects, such as an increase in local perfusion and enhancement of neural function, which correlated with improved cognitive function.

The current systematic review aims to summarize the techniques (FUS exposure parameters, treatment sessions, BBB opening assessment and side effects) employed in transcranial FUS applications in preclinical animal models and in humans, the possible mechanisms underlying FUS therapeutic effects on AD pathology and cognitive impairment, as well as the current limitations and challenges of FUS treatment on AD. This review should provide useful information for future clinical applications.

## MATERIALS AND METHODS

For this systematic review, we followed the Preferred Reporting Items for Systematic Reviews and Meta-Analyses (PRISMA) criteria [29]. Electronic searches were conducted on the main biomedical databases PubMed, MEDLINE, Web of Science and EMBASE from 2001 to 2020. The following keywords were used: “Focused ultrasound”, “low-intensity pulsed ultrasound”, “transcranial ultrasound”, “scanning ultrasound”, “Alzheimer’s disease”, “amyloid β” and “tau”. Additional searches used Google Scholar search tools and the reference list of relevant reviews.

### Inclusion and exclusion criteria

We followed the Population, Intervention, Comparison, Outcomes and Study (PICOS) design as a framework to establish inclusion criteria. Studies under the following inclusion criteria were selected: (1) Population (P): studies that used AD animal models or AD patients as the experimental subjects; (2) Intervention (I): studies that used transcranial focused ultrasound (tFUS), MRI guided focused ultrasound (MRIgFUS), scanning ultrasound (SUS), low-intensity pulsed ultrasound (LIPUS) with or without infusion of microbubbles to perform drug delivery or treatment; (3) Comparison (C): studies that compared AD vs control groups, and FUS treatment vs sham groups; Outcomes (O): studies that provided at least one outcome measurement evaluating BBB opening, efficacy of drug delivery, reduction of Aβ or tau burden, neurogenesis, neural plasticity or angiogenesis, enhancement of neural function (neural activity and functional connectivity), increased cholinergic function and improvement of cognitive or memory impairment through immunofluorescence histochemical staining, neuroimaging (*e.g.* MRI, PET) and neurobehavioral tests in cognition and memory domains; (4) Study design (S): Randomized controlled or non-randomized controlled studies or clinical trials or case reports; (5) Original articles; (6) Published in the English language.

Studies meeting any of the following criteria were excluded: (1) Review articles, editorials, journal reports, theses, and expert opinion or commentary; (2) Conference materials and abstracts; (3) FUS induced BBB opening not used for drug delivery or treatment.

### Data extraction

After finalizing the inclusion articles, two authors (XDL and NSS) independently extracted the following information from each article: (1) authors and publication year; (2) types of experimental animals; (3) types of FUS (FUS-MB with drug delivery, FUS-MB treatment, FUS stimulation); (4) FUS parameters (central frequency of transducer, acoustic pressure, pulse scheme, sonication duration, single or repeated treatment) and types and dose of MB; (5) target sites; (6) assessment of BBB opening; (7) adverse effects; (8) main findings; (9) mechanisms of therapeutic effects by FUS. For drug delivery studies, we also summarized the types of the drugs and pharmacological mechanisms. For human studies, we summarized the FUS parameters, side effects and outcomes of FUS application.

### Methodological quality assessment of included studies

The Systematic Review Center for Laboratory Animal Experimentation risk of bias (SYRCLE’s RoB) tool [30] was used to assess risk of bias in the animal studies. The SYRCLE’s RoB tool consists of 10 items that are related to selection bias, performance bias, detection bias, reporting bias and other biases. Two authors (XDL and NSS) independently conducted the assessment. The Physiotherapy Evidence Database (PEDro) scale [31] was used to assess the included human randomized controlled trials. The PEDro scale consists of 11 items including eligibility criteria, random allocation, concealment of allocation, baseline equivalences, blinding, outcome measures, between-group statistical comparisons, point and variability measures. Disagreements were solved through consensus by a third author (REJ).

## RESULTS

### Characteristics of studies

**Figure 1.**
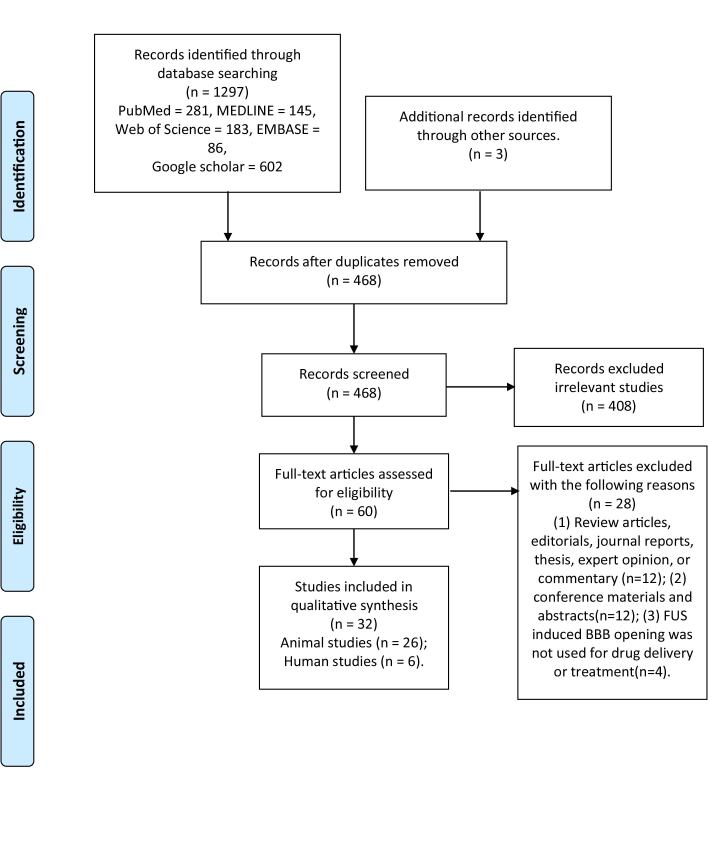
**Selection process for the studies included in this review.** The scheme is from Ref [29].

The review and selection of studies process is shown in the PRISMA Flow diagram (Fig. 1). Briefly, the initial search retrieved 1,297 manuscripts. After removing duplicates, the remaining 468 articles were further screened by reading the title and abstract, of which 408 articles were excluded because they were irrelevant. A total of 60 articles were subjected to full-text review, of which 28 articles were removed based on the exclusion criteria. Ultimately 32 studies were selected for this review, including 26 animal studies and 6 human studies.

The methodological quality of included animal studies assessed by the SYRCLE showed 55% of items classified as “unclear” and 0.3% of items classified as “no”. The average PEDro score for 4 human studies was 6.5/11. Summarized information is provided in Tables 1 and 2.

### FUS applications in AD animal models

#### FUS-MB with drug delivery

A total of 12 animal studies regarding drug delivery using FUS-MB were reviewed. The relevant information is shown in Table 3. In these studies, we found that MRI guided FUS (MRIgFUS) was most commonly used for drug delivery. Scanning ultrasound (SUS) was often applied to target large anatomic areas, such as the forebrain or the entire brain. Gadolinium enhanced MRI and Trypan blue/Evans blue dye were used for confirming the extent of BBB opening. T2 weighted MR imaging (T2WI), susceptibility weighted imaging (SWI) and histological staining (hematoxylin-eosin (H&E), Prussian blue, Nissel and acid fuchsin) were used to assess tissue damage (hemorrhage, edema and neuronal degeneration and loss). The most important FUS parameters for the safety and efficacy include the central frequency of the transducer (0.5-1.7 MHz) and the acoustic pressure. Acoustic pressure of 0.3-0.67 MPa was shown to disrupt BBB without obvious neuronal cell death or bleeds. Raymond et al. [32] and Alecou et al. [6] reported that acoustic pressure of 0.67MPa and 0.8MPa resulted in small hemorrhages observed in H&E staining. Several research groups further utilized passive cavitation detection (PCD) of MB to control the acoustic pressure in a safe range [8, 11, 13, 14]. When sub-harmonic emission was detected, the acoustic pressure amplitude was adjusted to a certain threshold and maintained for the rest of sonication duration. A pulse scheme with 10 ms pulse length, 1 Hz pulse repetition frequency (PRF) for 120s per spot was consistent across most studies. SUS studies used higher PRF (10 Hz) with shorter duration (6s per spot), because SUS was used to targeted multiple spots (20-24 sports) during a single sonication session. Commercial MB, such as Optison, Definity, and SonoVue, and custom-made MBs were introduced to assist with the BBB opening, but the concentration and dose were not consistent across studies. Most drug delivery studies used a single FUS-MB session. Alecou et al. [6] compared a single session with multiple sessions (2-3 sessions) of FUS-MB treatment and found multiple sessions with anti-Aβ antibody (BC-10) enhanced the effects on the reduction of Aβ burden. Several other studies also employed repeated SUS-MB and FUS-MB treatment to deliver larger therapeutic agents (e.g. anti-tau antibodies (29 kDa-156 kDa) and glycogen synthase kinase (GSK)-3 (308 kDa)) and nanoparticles (Qc@SNPs), demonstrating excellent therapeutic effects [7, 9, 12].

The included studies showed that FUS-MB induced BBB opening allowed permeation of various large therapeutic agents. Four research groups showed that single FUS-MB treatment facilitated anti-Aβ antibodies (A8326, BAM-10 and BC-10) and intravenous immunoglobulin (IVIg) entering the brain and binding to Aβ plaque, consequently lowered Aβ plaque burden in the targeted regions [5, 6, 14, 32]. Liu et al. [8] further found that scyllo-inositol (SI) in addition to BAM-10/FUS-MB saturated the early benefit of BAM-10/FUS-MB, due to SI stabilized small soluble conformers of Aβ that are cleared by microglia. Nisbet et al. [7] and Janowicz et al. [33] reported repeated SUS-MB treatment enhanced anti-tau antibody (RN2N) delivery to neurons, regardless of the antibody format, and significantly reduced phosphorylated tau (p-tau) levels. Hsu et al. [9] demonstrated that repeated FUS-MB enhanced the entry of GSK-3 inhibitor, which had an additive effect on Aβ plaque reduction. Xhima et al. [13] observed that MRIgFUS effectively delivered TrkA agonist D3 to basal forebrain, which led to TrkA signaling in cholinergic neurons (BFCNs) and elevated choline acetyltransferase (ChAT) activity and acetycholine (Ach) release, therefore rescuing cholinergic function. In addition, Xu et al. [10] and Liu et al. [12] found that FUS-MB could facilitate brain entry of nanoparticles (*i.e.* protoporphyrin IX (PX)-modified oxidized mesoporous carbon nanospheres (PX@OP@RVGs), quercetin-modified sulfur nanoparticles (Qc@SNPs) and assisted in the effective release of components from nanocarriers into target regions, resulting in reduction of Aβ plaque, p-tau and neuronal loss as well as improvement of memory and cognitive function. Furthermore, Weber-Adrain, et al. [11] illustrated that FUS-MB treatment was of benefit for gene therapies by allowing the gene vector (recombinant adeno-associated virus mosaic serotype (rAAV1/2) with glial fibrillary acidic protein (GFAP) promoter (rAAV1/2-GFAP) or human beta actin promoter (rAAV1/2-HBA)) to enter the brain and regulate transgene expression.

**Table 1:** Quality assessment of included animal studies by SYRCLE’s tool.

Study	Selection bias			Performance bias		Detection bias		Attrition bias	Reporting bias	Other
	Sequence generation	Baseline characteristics	Allocation concealment	Random housing	Blinding	Random outcome assessment	Blinding	Incomplete outcome data	Selective outcome reporting	Other sources of bias
**Raymond et al 2008**	unclear	yes	unclear	unclear	unclear	yes	unclear	yes	yes	yes
**Jardao et al 2010**	unclear	yes	unclear	unclear	unclear	yes	unclear	yes	yes	yes
**Jardao et al 2013**	unclear	yes	unclear	unclear	unclear	yes	unclear	yes	yes	yes
**Burgess et al 2014**	unclear	yes	unclear	unclear	unclear	yes	unclear	yes	yes	yes
**Lin et al 2015**	unclear	yes	unclear	unclear	unclear	yes	unclear	yes	yes	yes
**Leinenga et al 2015**	unclear	yes	unclear	unclear	yes	yes	unclear	yes	yes	yes
**Alecou et al 2017**	unclear	yes	unclear	unclear	unclear	yes	unclear	yes	yes	yes
**Nisbet et al 2017**	unclear	yes	unclear	unclear	unclear	yes	unclear	unclear	yes	yes
**Li et al 2017**	unclear	yes	unclear	unclear	unclear	yes	unclear	unclear	yes	yes
**Liu et al 2018**	unclear	yes	unclear	unclear	unclear	yes	no	unclear	yes	yes
**Hsu et al 2018**	unclear	yes	unclear	unclear	unclear	yes	unclear	yes	yes	yes
**Xu et al 2018**	unclear	yes	unclear	unclear	unclear	yes	unclear	unclear	yes	yes
**Leinenga et al 2018**	unclear	unclear	unclear	unclear	unclear	yes	unclear	yes	yes	yes
**Poon et al 2018**	unclear	yes	unclear	unclear	unclear	yes	unclear	unclear	yes	yes
**Eguchi et al 2018**	unclear	yes	unclear	unclear	unclear	yes	unclear	unclear	yes	yes
**Janowicz et al 2019**	unclear	yes	unclear	unclear	unclear	yes	unclear	unclear	yes	yes
**Weber-Adrian et al 2019**	unclear	yes	unclear	unclear	unclear	yes	unclear	unclear	yes	yes
**Karakatsani et al 2019**	unclear	yes	unclear	yes	unclear	yes	unclear	unclear	yes	yes
**Leinenga et al 2019**	unclear	yes	unclear	unclear	unclear	yes	unclear	yes	yes	yes
**Pandit et al 2019**	unclear	yes	unclear	unclear	unclear	yes	unclear	unclear	yes	yes
**Shin et al 2019**	unclear	yes	unclear	unclear	unclear	yes	unclear	unclear	yes	yes
**Liu et al 2020**	unclear	unclear	unclear	unclear	unclear	yes	unclear	unclear	yes	yes
**Xhima et al 2020**	unclear	yes	unclear	unclear	unclear	yes	yes	unclear	yes	yes
**Lee et al 2020**	unclear	yes	unclear	unclear	unclear	yes	unclear	unclear	yes	yes
**Bobola et al 2020**	unclear	yes	unclear	unclear	unclear	yes	unclear	unclear	yes	yes
**Dubey et al 2020**	unclear	yes	unclear	unclear	unclear	yes	unclear	unclear	yes	yes

**Table 2:** Quality assessment of included human studies by PEDro.

Study	Eligiblety criteria	Random allocation	Concealed allocation	Baseline comparability	Blind subjects	Blind therapists	Blind assessors	Adequate follow-up	Intention-to-treat analysis	Between group comparisons	Point estimates and variability	Total scores
**Lipsman et al 2018**	1	0	0	1	1	0	0	1	1	1	1	7/11
**Meng et al 2019**	1	1	0	1	1	0	0	1	1	1	1	8/11
**Beisteiner et al 2019**	1	0	0	1	1	0	0	1	1	1	1	7/11
**Nicodemus et al 2019**	1	0	0	1	1	0	0	1	1	0	1	6/11
**Meng et al 2019**	1	0	0	0	1	0	0	1	0	0	1	4/11
**Rezai et al 2020**	1	0	0	1	1	0	0	1	1	1	1	7/11

### FUS-MB treatment

A total of 9 animal studies using FUS-MB solely as treatment without therapeutic agents were included. Relevant information is shown in Table 3. The FUS parameters and MB type and dose for safe BBB opening were similar to those used in FUS-MB with drug delivery. Repeated FUS-MB treatment (weekly or biweekly for a total of 4-10 weeks) were more commonly used compared to drug delivery and the treated animals did not present obvious short-term side effects with the FUS parameters employed. Poon, et al. [18] further reported that repeated MRIgFUS-MB treatment was more effective for reducing Aβ pathology compared to a single intracranial FUS-MB treatment.

Most included studies revealed that both a single and repeated FUS-MB treatment that induced BBB opening allowed the entry of endogenous immunoglobulin (IgG and IgM) and activated glial cells, which presumably reduced Aβ plaque and p-tau burden and consequently rescued memory and cognitive deficits [15-17, 19, 34, 35]. Leinenga et al. [17] further found that repeated SUS-MB treatment could break down larger plaques into smaller plaque, facilitating Aβ uptake by microglia. Two studies showed repeated FUS-MB induced BBB opening also allowed the entry of peripheral immune cells aiding in Aβ plaque and p-tau clearance [18, 19]. Karakatsani et al. [19] further observed that immune cells and microglia could migrate to non-sonicated regions to exert their effects. Leinenga et al. [34] reported that repeated SUS-MB treatment did not induce an inflammatory response associated with tissue damage. Pandit et al. [20] and Lee et al. [22] found that repeated FUS-MB induced BBB opening enhanced clearance of Aβ and p-tau through an autophagy mediated pathway and glymphatic-lymphatic pathway. Furthermore, two studies illustrated that FUS-MB treatment increased neuronal plasticity and neurogenesis in the hippocampus [16, 36]. Shin et al. [36] identified FUS-MB treatment leading to the recovery of cholinergic function, which is critical for upregulating proliferation and neurogenesis and maintaining memory and cognitive function.

**Table 3:** Summary of the FUS applications in AD rodent model.

Refs.	Experimental animals	Protocol of FUS	Brain targets	BBB opening confirmation	Side effects after FUS	Major findings	Underlying mechanisms of therapeutic effects
FUS-MB with drug delivery							
**Raymond et al. (2008)**	11-12 months old B6C3-Tg (APPswe, PSEN1dE9) 85Dbo/J mice (n=2)	Type: MRIgFUS Frequency: 0.69 MHz Peak negative pressure: 0.67-0.8 MPa Burst length: 10ms Repetition frequency:1Hz Sonication duration: 40-45s MB: Optison 0.03-0.05ml or Definity (1:10 dilution) 0.01ml Single treatment	Right hippocampus	(1) Enhancement appeared at the targeted site on Gadolinium-based-contrast MRI (2) Trypan blue or Evans blue extravasation	2 mice treated at high pressure (0.8MPa) and 1 of 7 treated at a lower pressure (estimated 0.67MPa) had petechiae on H&E-stained section.	(1) Endogenous IgG extravasated across the BBB after MRIgFUS-MB treatment (2) Anti- Aβ antibodies (A8326) were delivered and heterogeneous distributed in the treated hemisphere after MRIgFUS-MB treatment.	MRIgFUS-MB treatment allows endogenous IgG and anti-Aβ antibodies to enter the brain, facilitates endogenous IgG and anti-Aβ antibodies binding to Aβ plaques and clearing the plaques
**Jordao et al. (2010)**	4-5 months old TgCRND8 mice\ Untreated group (n=5) BAM-10/FUS group (n=6)	Type: MRIgFUS Frequency: 0.558 MHz Acoustic pressure: 0.3MPa Burst length: 10ms Repetition frequency:1Hz Sonication duration: 120s MB: Definity 0.16 ml/kg Single treatment	Right hemisphere	Enhancement appeared at the targeted site on Gadolinium-based-contrast MRI	No red blood cells were detected using Prussian blue staining	(1) BAM-10 anti-Aβ antibody was found bound to Aβ plaques only on the MRIgFUS-MB targeted side (2) BAM-10/MRIgFUS-MB treatment reduced Aβ plaque burden (size and total surface area)	MRIgFUS-MB treatment allows anti-Aβ antibody to enter the brain and facilitates anti-Aβ antibodies binding to Aβ plaques
**Alecou et al. (2017)**	2% high-cholesterol diet New Zealand White rabbits FUS (n=2) FUS+ antibodies (n=18) 3 sessions of FUS (n=4 per session) 3 sessions of FUS+ antibodies (n=4 per session)	Type: MRIgFUS Frequency: 1 MHz Acoustic pressure: 0.8MPa Burst length: 10ms Repetition frequency:1Hz Sonication duration: 20s MB: SonoVue 0.05 ml/kg Single treatment and 3 repeated sessions with 3 days apart	Right hemisphere	Enhancement appeared at the targeted site on Gadolinium-based-contrast MRI	Small hemorrhage detected on H&E-stained section	(1) BC-10 anti-Aβ antibody/MRIgFUS-MB treatment reduced Aβ plaque load in the targeted hemisphere (2) Multiple sessions of BC-10 anti-Aβ antibody/MRIgFUS-MB treatments enhanced the reduction of Aβ plaque compared to single treatment.	(1) MRIgFUS-MB treatment allows the anti-Aβ antibody to enter the brain and facilitates anti-Aβ antibody binding to Aβ plaques. (2) Repeated sessions of MRIgFUS-MB treatments enhance the therapeutic effects of anti-Aβ antibody.
**Nisbet et al. (2017)**	pR5 mice pR5 with SUS (n=6) pR5 with RN2N+SUS (n=5)	Type: SUS Frequency: 1 MHz Acoustic pressure: 0.7MPa Burst length: 10ms Repetition frequency:10Hz Duty cycle: 10% Sonication duration: 6s per spot MB: in-house Lipid-shelled MB 0.03ml Treated weekly for 4 weeks	Entire brain	Not mentioned	Not mentioned	(1) SUS-MB alone and RN2N/SUS-MB treatments reduced phosphorylated tau levels (2) RN2N/SUS-MB treatment reduced anxiety (3) RN2N treatment inhibited GSK3-mediated tau phosphorylation in vitro (4) SUS-MB treatment enhanced RN2N delivery across the BBB to neuron	(1) Repeated SUS-MB treatments allow RN2N to enter the neurons and facilitates RN2N binding to tau, preventing the interaction between GSK3β and tau required for phosphorylation. (2) Repeated SUS-MB treatments increase the turnover of phosphorylated tau through the ubiquitin pathway within neurons.
**Liu et al. (2018)**	5 months old TgCRND8 (n=6)	Type: MRIgFUS Frequency: 0.5515 MHz Acoustic pressure: based on the analysis of MB signal recorded during each burst Burst length: 10ms Repetition frequency:1Hz Sonication duration: 120s MB: Definity 0.04ml/kg Single treatment	Bilateral cortex	Not mentioned	Not mentioned	(1) Both SI and BAM10+SI/MRIgFUS-MB treatments significantly reduced Aβ load as well as increased microglial phagocytosis (2) SI treatment saturated the early benefit of the BAM-10/MRIgFUS-MB treatment (3) Both SI and BAM10+SI/MRIgFUS-MB treatments significantly reduced astrogliosis.	(1) BAM-10 targets the amyloid plaques and drives direct clearance by plaque associated microglia, whereas SI stabilizes small soluble conformers of Aβ that are phagocytosed by both plaque associated, and non-plaque associated microglia. (2) MRIgFUS-MB treatment allows the entry of BAM-10, thus either synergistically or additively increases the SI induced clearance of Aβ
**Hsu et al. (2018)**	12-14 months old APPswe/PSEN1-dE9 mice FUS (n=6) FUS+GSK-3 inhibitor (n=9)	Type: FUS Frequency: 0.4 MHz Acoustic pressure: 0.41-0.5MPa Burst length: 10ms Repetition frequency:1Hz Sonication duration: 60s MB: SonoVue 0.01ml 7days/ exposure for a total 5 times.	Right hippocampus	(1) Enhancement appeared at the targeted site on Gadolinium-based-contrast MRI (2) Evans blue extravasation	Not mentioned	(1) Repeated FUS-MB treatments enhanced GSK-3 inhibitor (AR-A014418) delivery into the brain and significantly reduced GSK-3 distribution. (2) Repeated AR/FUS-MB treatments significantly reduced Aβ	(1) GSK-3 links to Aβ production, tau phosphorylation and neuroinflammation (2) GSK-3 inhibitor was delivered by repeated FUS-MB treatments had an additive effect on plaque reduction.
**Xu et al. (2018)**	11 months old APP/PS1 mice OP@RVGs+FUS (n=12) APP/PSI mice with PX@OP@RVG+FUS (n=12)	Type: FUS Frequency: 1 MHz Sonication duration: 180s Single treatment	Brain	Not mentioned	Not mentioned	Nanoparticles (PX@OP@RVG) was delivered into the brain by FUS treatment reduced Aβ plaque and phosphorylated tau and thus rescued memory deficits.	(1) FUS treatment assists with nanoparticle release of PX into the brain. (2) PX leads to the production of ROS, which strongly suppresses Aβ aggregation, GSK3β and Aβ mediated phosphorylation of tau protein
**Janowicz et al. (2019)**	3-6 months old pR5 mice IgG+SUS group (n=5) Fab+SUS group (n=5) scFv+SUS group (n=5)	Type: SUS Frequency: 1 MHz Acoustic pressure: 0.65MPa (for whole brain)/0.6MPa (for hippocampus) Burst length: 10ms Repetition frequency:10Hz Duty cycle: 10% Sonication duration: 6s per spot (for whole brain)/60s (for hippocampus) MB: in-house Lipid-shelled MB 0.04ml	Whole brain/ hippocampus	Not mentioned	Not mentioned	SUS-MB treatment enhanced RN2N anti-tau antibody delivery to the brain regardless of antibody formats at the sonication site	SUS-MB treatment can deliver various formats of anti-tau antibody to the brain
**Weber-Adrian et al. (2019)**	3 months old TgCRND8 mice FUS+AAV1/2-GFAP-GFP (n=4) FUS+AAV1/2-HBA-GFP (n=4)	Type: MRIgFUS Frequency: 1.68 MHz Acoustic pressure: When a 840Hz sub-harmonic was detected, the pressure amplitude was dropped to 50% of the value ana maintained for the remainder of sonication duration Burst length: 10ms Repetition frequency:1Hz Sonication duration: 120s MB: Definity 0.02ml/kg Single treatment	Cortex and hippocampus	Enhancement appeared at the targeted site on Gadolinium-based-contrast MRI	Not mentioned	(1) MRIgFUS-MB treatment facilitated rAAV1/2 delivery to targeted regions. (2) GFAP and HBA promotors resulted in comparable numbers of GFP-positive cells (3) GFP expression under the GFAP promoter was enhanced near Aβ plaque. (4) GFAP promoter limited transgene expression in periphery	MRIgFUS-MB treatment allows the gene vector to enter the brain and regulates transgene expression near Aβ plaque
**Liu et al. (2020)**	6 months old APP/PS1 mice (n=3)	Type: FUS Sonication duration: 600s MB: poly (α-cyanoacrylate n-butyl acrylate)-based MB Repeated treatment for 5 weeks	Brain	Not mentioned	Not mentioned	(1) Qc@SNPs with FUS-MB treatment successfully promoted local BBB opening and conveyed Qc@SNPs into the brain. (2) Qc@SNPs/FUS-MB treatment, cognitive levels were improved, Aβ content and neuron loss were reduced	(1) FUS-MB treatment promotes nanoparticle release of Qc into the brain. (2) Qc has anti-oxidation and anti-inflammatory activity. (3) Qc can reduce neuronal apoptosis and Aβ content by reducing ER stress in the cell
**Xhima et al. (2020)**	6 months old TgCRND8 mice(n=5) Non-Tg mice (n=5)	Type: MRIgFUS Frequency: 1.68 MHz Acoustic pressure: When sub-harmonics were detected, the pressure amplitude was dropped to 25% of the value and maintained for the remainder of sonication duration. Burst length: 10ms. Repetition frequency:1Hz Sonication duration: 120s MB: Definity 0.02ml/kg Single treatment	Bilateral basal forebrain	(1) Enhancement appeared at the targeted site on Gadolinium-based-contrast MRI (2) Evans blue extravasation	No evidence of erythrocyte extravasation or neuronal cell death in sonicated area and no red blood cell infiltration into the brain parenchyma	(1) MRIgFUS-MB treatment effectively delivered TrkA agonist D3 (2) D3/MRIgFUS-MB treatment activated TrkA-dependent signaling pathways (3) D3/MRIgFUS-MB treatment enhanced cholinergic function	MRIgFUS-MB treatment facilitates the delivery of D3, leading to selective TrkA signaling in BFCNs while reducing p75^NTR^ activation, and elevating ChAT activity and Ach release.
**Dubey et al 2020**	(1) Bioavailability: 3-4 months old TgCRND8 mice (n=24) Non-Tg (n=24) (2) Efficacy: 3-4 months old TgCRND8 mice (n=59) Non-Tg (n=68)	Type: MRIgFUS Frequency: 1.68 MHz Acoustic pressure: the sonication was controlled by a feedback controller and allowed for consistent BBB permeabilization. Burst length: 10ms. Repetition frequency:1Hz Sonication duration: 120s MB: Definity 0.02ml/kg Single treatment (Bioavailability study); Weekly treatment for two weeks (Efficacy study)	(1)Bioavailability study: left hippocampus and frontal cortex (2) Efficacy study: bilateral hippocampus	Enhancement appeared at the targeted site on Gadodiamide-based-contrast MRI	Not mentioned	(1) Bioavailability: one administration of IVIg-FUS-MB treatment delivered 0.09% (Tg) and 0.06% (nTg) of the injected dose to the targeted regions at 4h and remained elevated at 24h post-FUS-MB treatment. (2) Efficacy: a. The detection of Ig-immunoreactivity in FUS-MB targeted hippocampi was higher compared to animals that received IVIg alone and saline. b. All plaque pathology was reduced by all treatments (IVIg, FUS and IVIg-FUS) c. FUS-MB treatment was required for IVIg to promoted hippocampal neurogenesis. d. FUS-MB treatment decreased hippocampal TNF-α	(1) IVIg exerts its effects by : a) binding to aggregating and pathological forms of Aβ and tau; b) promoting the efflux of Aβ from brain; c) engaging immune-mediated responses involved in the clearance of Aβ; d) attenuating cell-death pathway and protecting neurons against Aβ; e) acting as an immunomodulator potentially through FcγRIIB and sialylated Fc. (2) FUS-MB treatment enhances the efficacy of IVIg by reducing AD pathology and promoting neurogenesis in hippocampus. (3) FUS-MB treatment decreases proinflammatory TNF-α, known to inhibit neurogenesis and influences Aβ pathologies and cognitive deficits.
FUS-MB treatment							
**Jordao et al. (2013)**	4 months old TgCRND8 mice (n=20) Non-Tg (n=21)	Type: MRIgFUS Frequency: 0.5 MHz Acoustic pressure: 0.3MPa Burst length: 10ms Repetition frequency:1Hz Sonication duration: 120s MB: Definity 0.08 ml/kg Single treatment	Right cortex	Enhancement appeared at the targeted site on Gadolinium-based-contrast MRI	Not mentioned	(1) MRIgFUS-MB treatment reduced Aβ plaque burden (size and total surface area) in targeted cortical regions (2) MRIgFUS-MB treatment allowed endogenous immunoglobulin (IgG and IgM) to enter the brain (3) Time dependent increased microglia and astrocyte activation surrounding Aβ plaque were found after MRIgFUS-MB treatment	(1) MRIgFUS-MB treatment facilitates the entry of endogenous antibodies and activates glial cells in the brain, (2) Antibodies invading the targeted area contribute to Aβ plaque reduction (2) Activated microglia and astrocytes can internalize Aβ and facilitate its clearance by solubilization of Aβ
**Burgess et al. (2014)**	7 months old TgCRND8 mice (n=8) Non-Tg (n=8)	Type: MRIgFUS Frequency: 1.68 MHz Acoustic pressure: When sub-harmonic emissions were detected, the acoustic pressure was reduced to half and maintained for the remainder of sonication duration Burst length: 10ms Repetition frequency:1Hz Sonication duration: 120s MB: Definity 0.02ml/kg Treated weekly for 3 weeks	Hippocampus	Enhancement appeared at the targeted site on Gadolinium-based-contrast MRI	(1) Animal’s weight, grooming or other activities related to general health were not affected (2) No histologic signs of tissue damage	(1) Repeated MRIgFUS-MB treatments improved cognitive performance (2) Repeated MRIgFUS-MB treatments reduced Aβ plaque load (3) Repeated MRIgFUS-MB treatments increased the proliferation and maturation of neurons in the targeted hippocampus, which was correlated with improved spatial memory function.	(1) Repeated MRIgFUS-MB treatments permit the entry of endogenous immunoglobulin from the periphery into the brain, which assists with plaque clearance. (2) Repeated MRIgFUS-MB treatments cause activation of astrocytes and microglia which internalizes amyloid and contribute to plaque reduction. (3) Repeated MRIgFUS-MB treatments increase production of BDNF which mediates neural plasticity in the hippocampus (4) Repeated MRIgFUS-MB treatments induce Akt signaling leading to increased survival of immature neurons.
**Leinenga et al. (2015)**	12-13 months old APP 23 mice (n=10)	Type: SUS Frequency: 1MHz Acoustic pressure: 0.7 MPa Burst length: 10ms Repetition frequency:10Hz Duty cycle: 10% Sonication duration: 6s per spot MB: in-house Lipid-shelled MB 0.001ml/g Treated weekly for 6 or 7 weeks	Entire brain	Evans blue extravasation	No neuronal degeneration on Nissel staining or edema or erythrocyte extravasation on H&E staining	(1) Repeated SUS-MB treatments reduced Aβ plaque load (2) Repeated SUS-MB treatments induced microglial activation (3) Repeated SUS-MB treatments restored memory function (4) Repeated SUS-MB treatments did not upregulate inflammation markers associated with tissue damage	(1) Repeated SUS-MB treatments cause uptake of Aβ into microglia lysosomes (2) Repeated SUS-MB treatments allow albumin entering the brain, which binds to Aβ and facilitates Aβ uptake by microglia
**Leinenga et al. (2018)**	21-22 months old APP23 mice (n=5)	Type: SUS Frequency: 1MHz Acoustic pressure: 0.7 MPa Burst length: 10ms Repetition frequency:10Hz Duty cycle: 10% Sonication duration: 6s per spot MB: in-house Lipid-shelled MB 0.001ml/g Treated biweekly for 8 weeks	Entire brain	Not mentioned	Microbleeds were found on the H&E staining in one of five SUS-treated mice	(1) Repeated SUS-MB treatments reduced the fraction of larger plaques, but not the total plaque area (2) Repeated SUS-MB treatments reduced fibrillar amyloid (3) Repeated SUS-MB treatments increased the number of plaque-associated microglia (4) Repeated SUS-MB treatments caused reductions in amyloid pathology even at an advanced stage.	(1) Repeated SUS-MB treatments break down the larger plaques into smaller plaques as the microglia perform their role of taking up Aβ (2) The englobement and degradation of large plaques are based on the increased number of plaque-associated microglia activated by repeated SUS-MB treatments
**Poon et al. (2018)**	(1) Single treatment 6 months oldTgCRND8 (n=5) (2) Repeated treatment 6 months old TgCRND8 (n=13) Non-Tg (n=11)	(1) Type: Intracranial FUS Frequency: 1.1MHz In situ pressure: 0.4-0.8MPa Burst length: 10ms Repetition frequency:1Hz Sonication duration: 120s MB: Definity MB 0.04ml/kg Single treatment (2) Type: MRIgFUS Frequency: 1.68MHz Acoustic pressure: When sub-harmonic emissions reached a threshold of 3.5 times the magnitude of background signals, the acoustic pressure was reduced by 50% and maintained for the remainder of sonication duration Burst length: 10ms Repetition frequency:1Hz Sonication duration: 120s MB: Definity 0.02ml/kg Treated biweekly for 10 weeks	Dorsal hippocampus	(1)Enhancement appeared at the targeted site on Gadolinium-based-contrast MRI (2) The leakage of fluorescent dextran from blood vessels into the extravascular space observed by two-photon fluorescence microscopy	Not mentioned	(1) Single FUS-MB treatment significantly reduced Aβ plaque volume at two days post-sonication and persisted for two weeks (2) Repeated MRIgFUS-MB treatments had an additive effect in reducing plaque number and surface area in the targeted hippocampus.	(1) FUS-MB treatment allows the entry of endogenous immunoglobulins which binds to Aβ plaque (2) FUS-MB treatment induces activation and increases phagocytosis of Aβ in microglia and astrocytes, particularly in the microglial lysosomal compartment (3) FUS-MB treatment induces the infiltration of systemic phagocytic immune cells into the brain, which can aid in Aβ plaque clearance
**Karakatsani et al. (2019)**	3.5-4.5 months old rTg4510 mice (n=13)	Type: FUS Frequency: 1.5MHz Acoustic pressure: 0.45MPa Burst length: 6.7ms Repetition frequency:10Hz Sonication duration: 60s MB: in-house MB 0.0001ml/g Treated weekly for 4 weeks	Hippocampus	Enhancement appeared at the targeted site on Gadolinium-based-contrast MRI	(1) No evidence of edematous incidences on the T2-weighted images (2) No negative impact on the neuronal integrity	(1) Repeated FUS-MB treatments reduced phosphorylated tau (p-tau) from the hippocampal neuronal processes (2) Repeated FUS-MB treatments facilitated peripheral immune cells entering the brain and activates immune cells) which correlated with p-tau reduction (3) Repeated FUS-MB treatments increased microglia activity colocalized with p-tau in hippocampus (4) The bilateral reduction in p-tau resulted from unilateral repeated FUS-MB treatment	(1) Repeated FUS-MB treatments drive a “healthy” activation of microglia or infiltrating immune cells that help reduce p-tau (2) The presence of immune cells and their colocalization with p-tau in the contralateral-to-ultrasound hemisphere can be driven by the migration of the resident microglia from the sonicated hemisphere through the integrating tract and /or the infiltration of peripheral cells
**Pandit et al. (2019)**	K3 mice (n=10)	Type: SUS Frequency: 1MHz Acoustic pressure: 0.65MPa Burst length: 10ms Repetition frequency:10Hz Duty cycle: 10% Sonication duration: 6s per spot MB: in-house phospholipid-shelled MB 0.001ml/g Treated biweekly for 15 weeks	Entire brain	Not mentioned	Not mentioned	(1) Repeated SUS-MB treatments reduced hyperphosphorylated tau and neurofibrillary tangles (2) Repeated SUS-MB treatments induced autophagy-mediated clearance of tau (3) Repeated SUS-MB treatments improved locomotor and memory function	Repeated SUS-MB treatments induce autophagy specifically in neurons which contributes to tau clearance
**Shin et al. (2019)**	SAP treated rat (n=16)	Type: FUS Frequency: 0.5MHz Acoustic pressure: 0.25MPa Burst length: 10ms Repetition frequency:1Hz Sonication duration: 120s MB: Definity Single treatment	Bilateral hippocampus	Enhancement appeared at the targeted site on Gadolinium-based-contrast MRI	Not mentioned	(1) FUS-MB treatment reduced AChE activity in the frontal cortex and hippocampus (2) FUS-MB treatment increased mature-BDNF expression (3) FUS-MB treatment increased EGR1 expression (a marker of neuronal plasticity) and the number of DCX+ cells (marker of immature neurons) and BrdU+ cells (marker of neurons) in hippocampus (4) FUS-MB treatment improved memory and cognitive function	(1) FUS-MB treatment results in the recovery of ACh levels, which is critical for upregulating proliferative activity and subsequent neurogenesis (2) FUS-MB treatment can promote BDNF expression, which contributes to the hippocampal neurogenesis positively in cholinergic degeneration (3) FUS-MB treatment leads to an increase in BDNF, neuroplasticity and hippocampal neurogenesis, resulting in an improvement in cognitive function
**Lee et al. (2020)**	4 months old 5XFAD mice (n=14) Non-Tg (n=12)	Type: FUS Frequency: 0.715MHz Acoustic pressure: 0.42MPa Burst length: 20ms Repetition frequency:1Hz Duty cycle: 2% Sonication duration: 60s MB: SonoVue 0.1ml Treated weekly for 6 weeks	One-third of hemisphere	Evans blue extravasation	No neuronal loss	(1) Repeated FUS-MB treatments reduced Aβ deposits and ameliorated glial activation in the entire brain, as well as targeted regions (2) Repeated FUS-MB treatments increased solute Aβ to the CSF space (3) CSF Aβ drainage by repeated FUS-MB treatments via meningeal lymphatics (4) Repeated FUS-MB treatments improved the working memory (5) Repeated FUS-MB treatments was not found to reactive astrocytes and microglial propensity surrounding Aβ deposits	(1) Repeated FUS-MB treatments enhance clearance of Aβ via glymphatic-lymphatic system (2) Repeated FUS-MB treatments can affect AQP4, which facilitates waste disposal via glymphatic-lymphatic system (3) MB cavitation in the arteries might function to mimic and enhance arterial pulsatility driving the ISF-CSF efflux of Aβ solutes (4) Repeated FUS-MB treatments restore memory by increasing glymphatic-lymphatic clearance of amyloid
FUS stimulation							
**Lin et al. (2015)**	AlCl_3_ treated rats (n=6)	Type: LIPUS Frequency: 1MHz I_SPTA_: 0.528W/cm^2^ Burst length: 50 ms Repetition frequency:1Hz Duty cycle: 5% Sonication duration: 5 min Treated with triple sonication daily for 42 days	Right hemisphere		Not mentioned	(1) Repeated LIPUS stimulations enhanced the expressions of neurotrophic factors (BDNF, GDNF and VEGF) in stimulated hippocampus (2) Repeated LIPUS stimulations attenuated the increase in aluminum concentration and AChE activity (3) Repeated LIPUS stimulations attenuated the increase in Aβ_1-42_ expression (4) Repeated LIPUS stimulations alleviated learning and memory deficits (5) Repeated LIPUS stimulations ameliorated AlCl_3_ associated cerebral damages	Repeated LIPUS stimulations prevent Al overload-induced damage of learning and memory function, karyopyknosis, inhibits increased AChE activity, down-regulates the protein expression of Aβ content and increases neurotrophic factors, which aids with controlling or reversing AD.
**Li et al. (2017)**	4.5 months old APP/PS1 mice (n=10)	Type: FUS Frequency: 1MHz I_SPTA_: 0.3W/cm^2^ Sonication duration: 10 min Treated daily for 6 weeks	Entire brain		Not mentioned	(1) Repeated FUS stimulations improve the spatial learning and memory ability (2) Amyloid deposition was not found in the hippocampus of the repeated FUS stimulations group	(1) Repeated FUS stimulations can regulate neuronal activity and even promote the brain’s cognitive function (2) Repeated FUS stimulations can active glial cells, which swallow amyloid, thereby reducing amyloid plaques
**Eguchi et al. (2018)**	3 months old 5XFAD (n=18) WT (n=18)	Type: LIPUS Frequency: 1.875MHz I_SPTA_: 0.099W/cm^2^ Burst length: 0.017ms The number of cycles: 32 Sonication duration: 20 min Treated with triple sonication on days 1,3,5,28,30,32,56,58,60,84 and 86	Entire brain		(1) No effects on body weight or blood pressure. (2) Did not cause cramps, paralysis, cerebral hemorrhage, hypothermia, hyperthermia or death or hyperactivity	(1) Repeated LIPUS stimulations significantly improved cognitive function (2) Repeated LIPUS stimulations ameliorated the decline in CBF (3) Repeated LIPUS stimulations suppressed chronic inflammatory response of microglia and enhanced endothelium-related genes (4) Repeated LIPUS stimulations reduced microgliosis along with eNOS upregulation (5) Repeated LIPUS stimulations increased NGF and pro-BDNF	(1) Repeated LIPUSs stimulation ameliorate cognitive dysfunctions by reducing Aβ and microgliosis (2) The reduction in Aβ results from the decreased expression of APP and BACE-1, changes in characteristics of microglia and refolding of Aβ by Hsp 90 after chronic LIPUS stimulations (3) eNOS is upregulated by chronic LIPUS stimulations associated with activated glial cells, APP, BACE-1 and Hsp 90, contributing to Aβ reduction (4) Repeated LIPUS stimulations activate endothelial cells that may have effects on astrocytes, resulting in improvement of cognitive functions
**Leinenga et al. (2019)**	12-14 months old APP23 mice (n=6) WT (n=5)	Type: SUS Frequency: 1MHz Acoustic pressure: 0.7 MPa Burst length: 10ms Repetition frequency:10Hz Duty cycle: 10% Sonication duration: 6s per spot Treated weekly for 5 weeks	Half hemisphere		No bleeds and neuronal loss on H&E staining section	Repeated SUS stimulations did not significantly reduce amyloid load, including plaque size and plaque number	Repeated SUS stimulations are not sufficient in amyloid clearance, but may ameliorate reductions in synaptic activity
**Bobola et al. (2020)**	6 months 5XFAD mice (n=5)	Type: FUS Frequency: 2MHz I_SPTA_: 3.0 W/cm^2^ I_SPPA_: 190 W/cm^2^ Burst length: 0.4ms Repetition frequency:40Hz Duty cycle: 10% Sonication duration: 1h Single treatment / Repeated treatment daily for 4 days	Left hippocampus (Single treatment) Bilateral hippocampus (repeated treatment)		Not mentioned	(1) Neither repeated nor single FUS stimulation significantly increased production of eNOS (2) Single FUS stimulation activated microglia that colocalized with Aβ plaque in all recruited mice (3) Repeated FUS stimulations increased activated microglia that colocalized with Aβ plaque across the entire treated brain in 60% of the mice (3) Repeated FUS stimulations decreased Aβ load within treated hippocampus	The reduction in Aβ burden due to activation of microglia induced by FUS stimulations

BBB= blood-brain barrier; MRIgFUS = MRI-guided focused ultrasound; SUS = scanning ultrasound; LIPUS = low intensity pulsed ultrasound; H&E = hematoxylin and eosin; I_SPTA_ = spatial-peak temporal average intensity; I_SPPA_ = spatial peak pulse average intensity; GSK= Glycogen synthase kinase; SI= scyllo-inositol; PX@OP@RVG = protoporphyrin IX (PX)-modified oxidized mesoporous carbon nanospheres(OMCN)(PX@OMCN@PEG(OP)@RVGs); ROS= reactive oxygen species; rAAVs = recombinant adeno-associated viruses; GFP = green fluorescent protein; GFAP = glial fibrillary acidic protein; HBA = human beta actin; Qc@SNPs = quercetin-modified sulfur nanoparticles; BFCNs = basal forebrain cholinergic neurons; TrkA = tropomyosin receptor kinase A; p75NTR = p75 neurotrophin receptor; ChAT = choline acetyltransferase ; Ach = acetylcholine; SAP = selective immunotoxin 192 IgG-saporin; IVIg = intravenous immunoglobulin; BDNF = brain-derived neurotrophic factor; NGF = nerve growth factor; GDNF = glial cell line-derived neurotrophic factor; VEGF= vascular endothelial growth factor; AChE = acetylcholinesterase; CSF = cerebral spinal fluid; AQP4 = aquaporin-4; CBF = cerebral blood flow; eNOS = endothelial nitric oxide synthase; APP = amyloid precursor protein; BACE-1 = β-site APP-cleaving enzyme-1; Hsp 90 = heat shock protein 90

#### FUS stimulation

A total of 5 animal studies were included that used FUS as a method for brain stimulation. Relevant information is shown in Table 3. FUS stimulation protocols used higher frequency transducers (1-2MHz) compared to those used in FUS-MB induced BBB opening (<1MHz). Most FUS stimulation studies applied low intensity (I_SPTA_: 0.099w/cm2-0.528w/cm2) pulsed ultrasound (LIPUS) to target the whole brain, half of the brain (one hemisphere) or the hippocampus and demonstrated repeated LIPUS could lower Aβ burden[23-26]. Lin et al. [23] and Eguchi et al. [25] found repeated LIPUS could decrease the expression of Aβ peptide, thus attenuating the production of Aβ. One study by Leinenga et al. [37], however, found that repeated SUS treatment over the entire right hemisphere was not sufficient to induce Aβ clearance. Eguchi et al. [25] found that repeated LIPUS upregulated endothelial nitric oxide synthase (eNOS) associated with activated glial cells contributing to Aβ reduction. In addition, Bobola et al. [26] found that applying relatively higher I_SPTA_ (3 w/cm2) and 40 Hz repetition frequency FUS stimulation could directly induce microglia activation without an increase in eNOS. Furthermore, Lin et al. [23] and Eguchi et al. [25] observed that repeated LIPUS could increase cholinergic activity and expression of neurotrophic factors, thus increasing neurogenesis and alleviating memory and cognitive deficits.

**Table 4:** Summary of the FUS applications in AD patients.

References	Human subjects	Protocol of FUS	Brain targets	BBB opening confirmation	Side effects after FUS	Major findings
FUS-MB treatment						
**Lipsman et al. (2018)**	50-85 years old mild-to moderate AD patients (n=5)	Type: MRIgFUS Frequency: 220kHz Acoustic pressure: when sub-harmonic emissions detected, subsequent sonications were performed at 50% of this “cavitation threshold” power Burst length: 2ms on and 28ms off for 300ms Repetition interval:2.7s Duty cycle: 0.74% Sonication duration: 50s MB: Definity 0.004ml/kg Two treatment sessions with 1- month interval	Right frontal lobe (superior frontal gyrus white matter of the DLPFC) for stage 1 treatment as well as adjacent area for stage 2 treatment	Enhancement appeared at the targeted site on Gadolinium-based-contrast MRI	(1) Clinically, no patient experienced a serious adverse event during this study. 1 patient showed a transient increase in NPI-Q score and 1 patient experienced headache during follow-up. (2) Radiologically, no evidence of intracerebral hemorrhage or swelling. 2 patients had microhemorrhages which was resolved by the 24 h follow-up	(1) BBB was successfully opened in all patients who underwent the FUS procedure and restored at 24h following the procedure (2) No significant clinical changes (cognition or daily functioning) were detected at 3 months follow-up (3) Group [18F]-Florbetaben PET uptake changes in the ROIs were not statistically significant after stage 1 and 2 treatments compared to baseline
**Meng et al. (2019)**	AD patients (n=3)	Same as Lipsman’s study	Prefrontal lobe, hippocampus, anterior cingulate cortex, posterior parietal cortex and primary motor cortex	Enhancement appeared at the targeted site on Gadolinium-based-contrast MRI	No complications (e.g., hemorrhage)	(1) Increased BBB permeability to gadobutrol was demonstrated at all sonication targets regions. (2) FLAIR with contrast detected hyperintensity around multiple large cortical veins, including the veins of Labbe and Trolard that drain into the superior sagittal and transverse sinuses as well as the adjacent subarachnoid space of sonicated areas, suggesting glymphatic efflux persist persists following FUS-MB induced BBB opening in humans.
**Meng et al. (2019)**	Mean age of 66.8 years mild-to-moderate AD patients (n=5)	Same as Lipsman’s study	Right frontal lobe	Enhancement appeared at the targeted site on Gadolinium-based-contrast MRI	Not mentioned	(1) Increased BBB permeability in the sonicated regions. (2)A transient FC decrease within the ipsilateral FPN following MRIgFUS induced BBB opening, that recovered by the next day, suggesting MRIgFUS treatment may transiently affect neurologic function.
**Rezai et al. (2020)**	Early AD patients (n=6)	Type: MRIgFUS Frequency: 220kHz MB: Definity Three treatment sessions with 2 weeks interval	Hippocampus and EC	Enhancement appeared at the targeted site on Gadolinium-based-contrast MRI	All patients tolerated well, no treatment-related adverse effects or neurological changes up to 15 months after FUS-MB treatment T2* MRI following FUS-MB treatment and at subsequent follow-up did not indicate overt hemorrhage	(1) BBB opening was detected in the targeted hippocampus and resolved within 24h after FUS-MB treatment. (2) At 30 days after treatment, patients showed no clinically meaningful changes
FUS stimulation						
**Nicodemus et al. (2019)**	AD patients (n=11) and PD patients (n=11)	Type: FUS Frequency: 2MHz I_SPTA_: 0.520W/cm^2^ Sonication duration: 1h Treated weekly for 8 weeks	Mesial temporal lobe		All patients were able to tolerate treatment without notable side effects	(1) 63% patients had improvement in cognitive function following FUS treatment. (2) 9.1% patients demonstrated improvement in gross motor functioning (3) ASL images showed a greater that 50% increase in relative perfusion at the targeted regions after 1h FUS session.
**Beisteiner et al. (2020)**	AD patients (n=35)	Type: FUS Frequency: 2MHz Energy flux density: 0.2 mJ/mm^2^ Pulse repetition frequency: 5Hz Burst length: 3μs Pulse number: 6000 Sonication duration: 0.003ms Treated three sessions weekly for 2-4 weeks	(1) Dorsolateral prefrontal cortex and areas of the memory (including DMN) and language networks (2) global brain		During a 3-months follow-up period, patients did not show any relevant side effects. T2* and FLASH images did not reveal any hemorrhages, edema or any other type of new intracranial pathology	(1) Patient’s cognitive state were improved significantly after FUS treatment and remained stable over three months (2) Resting-state fMRI data showed upregulation of the memory network after FUS therapy that correlated with cognitive performance (3) Task-fMRI data confirmed activation increase in bilateral hippocampus after FUS treatment.

PD = Parkinson’s disease; DLPFC = dorsolateral prefrontal cortex; NPI-Q = Neuropsychiatric Inventory-Questionnaire; ROIs = regions of interest; FLAIR = fluid-attenuated inversion recovery; FC = functional activity; FPN = frontoparietal networks; EC = entorhinal cortex; DMN = default mode network; ASL = arterial spin labeling

### FUS applications in AD patients

#### FUS-MB treatment

A total of 4 human studies using FUS-MB treatment were recruited. Relevant information is shown in Table 4. These studies performed repeated MRIgFUS-MB (2-3 treatment sessions) on mild-to-moderate AD patients. The FUS parameters included a central frequency of 220kHz, sonication power of 4.5-4.6 W, 3.6-7.5 sonications for 300ms (each spot with 2ms on and 28ms off), and Definity MB infusion (4 μl/kg), which enabled BBB opening without obvious short- or long-term treatment-related side effects (e.g., death, hemorrhages, swelling, neurological deficits). Meng et al. [38] detected MRI hyperintensity within the perivascular space and subarachnoid space (SAS) on contrast enhanced fluid-attenuated inversion recovery (FLAIR) imaging after FUS-MB treatment, suggesting glymphatic efflux persists following FUS-MB induced BBB opening. They also found a transient decrease in functional connectivity (FC) within the ipsilateral frontoparietal networks (FPN) (restored within 24 h), indicating FUS-MB may transiently affect neuronal function [39]. Regarding the therapeutic effect of FUS-MB treatment on AD patients, Lipsman et al. [40] and Rezai et al. [41] showed there were no clinically meaningful changes (cognition or daily functioning) or changes in [18F]-Florbetaben PET uptake at 1- and 3-months follow-up in any AD subjects.

#### FUS stimulation

Two human studies using transcranial FUS stimulation were included. Relevant information is shown in Table 4. Nicodemus et al. [27] first reported the feasibility of transcranial FUS stimulation on AD and Parkinson’s disease (PD) patients. One-hour FUS stimulation was delivered using a 2MHz transducer at a power of 520mW/cm^2^ targeting the mesial temporal lobe guided by MRI and Doppler ultrasound. All the patients tolerated treatment without notable side effects. They found that 63% of patients had improvements in cognitive function and 9.1% of patients had improvements in gross motor functioning after 8 weeks’ FUS therapy. They also detected increased perfusion in the targeted region using arterial spin labeling (ASL) MRI [27]. Beisteiner et al. [28] reported a multicenter clinical trial using a single ultrashort ultrasound pulse stimulation to treat patients with probable AD. The FUS parameters: 0.2 mJ mm-2 energy flux density, 5Hz PRF, 6000 pulses per session and 3 μs pulse duration. The treatment comprised three sessions over 2-4 weeks and targeted the dorsolateral prefrontal cortex, memory areas (including default mode networks (DMN)) and language networks. All the AD subjects presented high treatment tolerability without relevant clinical side effects, tissue damages (*e.g*., hemorrhages and edema) or new intracranial pathology on MRI within a 3-month follow-up period. Clinical data showed that the patients’ cognitive state was improved after treatment and remained stable over three months. Functional MRI (fMRI) data demonstrated upregulation of memory network and hippocampus activation, which correlated with cognitive improvement in patients.

## DISCUSSION

This systematic review retrieved published studies in the past 12 years in both animals and humans that employed FUS for the treatment of AD. Currently, FUS application in AD can be categorized into the following: FUS-MB with drug delivery, FUS-MB treatment alone, and FUS stimulation.

### FUS-MB with drug delivery treatment

FUS with the infusion of MB has been regarded as a noninvasive approach that transiently opens the BBB to deliver therapeutic agents to the brain parenchyma. During the oscillating acoustic pressure, the MB undergoes stable cavitation (expansion and contraction without bursting) within the blood vessels at relatively low pressures. Because the MB is not much smaller than capillaries, mechanical effects likely perturb paracellular and transcellular barriers and immunosignals at tight junction proteins (*e.g*. occludin, claudin-5 and ZO-1) inducing BBB disruption [42, 43]. Electron microscopy has identified that therapeutic agents can pass through the disrupted BBB via transcellular and paracellular mechanisms, including transcytosis using cellular vesicles, endocytosis, paracellular passage through widened tight junction and through cytoplasmic channels in the endothelium[44]. Currently, there are no studies applying FUS-MB for drug delivery in AD patients. Animal studies demonstrated that FUS-MB induced BBB opening was able to permeate various therapeutic agents, including anti-Aβ and anti-tau antibodies (A8326, BAM-10, RN2N), IVIg, GSK-3 inhibitor (AR-A014418) and TrkA agonist (D3) with molecular weight up to 308 kDa. In addition, FUS-MB delivered a gene vector (rAAV1/2) enhancing local transgene expression and facilitated nanocarrier (Qc@SNPs and PX@OP@RVGs) release of effective components into targeted brain regions.

MRI-guided FUS system is the most commonly used technique applied in AD animal models, not only can MRI guide FUS to target precise regions, but MRI can also assess the extent of BBB opening and monitor side effects (*e.g*. hemorrhage and edema) after FUS exposure [45, 46]. SUS equipped with a motorized positioning system can move the transducer in small increments to cover large anatomic regions and is often used for whole brain drug delivery [7, 33]. FUS exposure parameters, including transducer frequency, acoustic pressure, pulse lengths, pulse repetition frequency, as well as the MB type and dose, are the main factors determining the safety and efficacy of FUS-MB induced BBB opening[45]. To confirm the extent of BBB opening and the drug delivery efficacy following FUS-MB, MRI and histological techniques can be used to visualize the extravasation of MRI gadolinium-based contrast agents and optical (Trypan blue or Evans blue) and fluorescently labeled dyes, respectively. Additionally, MRI (T2- and T2*-weighted imaging) and histological staining (hematoxylin and eosin (H&E), Nissel and acid fuchsin staining, anti-NeuN and anti-β-tubulin III staining) are employed to investigate the tissue damages (e.g., hemorrhages, edema, neuronal degeneration and loss). Compared to humans, AD animal models (rodents and rabbits) have a significantly thinner skull leading to reduced sonication power attenuation and thus use of overall lower power FUS to avoid tissue damages [45]. In rodent models, typically to open BBB in a safety manner without obvious tissue damages on MRI and histological staining sections, acoustic pressure ranged from 0.3MPa to 0.8MPa, with 10ms pulse lengths, 1-10 PRF and total duration of 20-120s are employed.

Previous studies have demonstrated that higher acoustic pressures will increase the BBB opening size, thus allowing bigger molecules to enter the brain. For example, Chen et al. [47] found that 0.31MPa allowed BBB opening for 3kD sized agents, while up to 70 kD entered at 0.51 MPa and up to 2000 kD at 0.84 MPa. However, they also detected that relatively smaller opening size (up to 70 kDa) was achieved with stable cavitation, while pressure required for larger opening sizes (above 500 kDa) caused inertial cavitation [47]. Inertial cavitation produces shock-waves or jets and has been associated with the extravasations of erythrocytes [48]. Two animal studies reported small hemorrhages detected in a few mice at 0.67 MPa and 0.8 MPa, indicating that inertial cavitation occurred and the acoustic pressure threshold for a safe BBB opening and drug delivery is <0.67MPa in the AD animal model [6, 32]. The passive cavitation detector (PCD) has been developed to monitor MB cavitation in real-time and provide feedback to the operator to adjust the acoustic pressure threshold [49]. Three animal studies applying PCD to control the acoustic pressure are noted [8, 11, 13]. Typically, transmit pressure is increased incrementally on a burst-by-burst basis until the sub-harmonics are detected, at which point the pressure is reduced and maintained for the duration of the experiment. Alternatively, repeated sonication can also enhance BBB permeability and prolong BBB opening [50]. Several studies have shown that repeated SUS-MB or FUS-MB treatment can enhance the permeability of relatively large therapeutic agents (*e.g*. anti-tau antibodies (29 kDa-156 kDa), IVIg (300 kDa) and glycogen synthase kinase (GSK)-3 (308 kDa)) and deliver the agents to neurons and exert excellent therapeutic effect on reducing the Aβ and tau load[7, 9, 12, 14, 33]. One of the included studies showed that 2-3 sessions of FUS-MB treatment could enhance the effects on the reduction of Aβ plaque when compared to a single treatment[6]. Optison, Definity and SonoVue are the most commonly used MB, however, the dose of these MBs is empirically determined depending on the goal of the study and varies across studies. A number of FUS studies used in-house custom-made MB to assist with the entry of anti-tau antibodies (RN2N) and nanoparticles (PX@OP@RVG) [7, 12, 33].

The studies reviewed show that FUS-MB induced BBB opening enhances the efficiency of drug delivery and improves the efficacy of treatment. Therapeutic effects depended on the pharmacological mechanisms of the drug itself, which included the following:

*Passive immunization: exogenous monoclonal anti-Aβ and anti-tau specific antibodies (BAM-10, BC-10 and RN2N) and IVIg.*

A number of included studies demonstrated that BAM-10, BC-10, various formats of RN2N and IVIg were delivered to targeted regions and neurons by a single or repeated FUS-MB treatment and bound to Aβ plaque and phosphorylated tau, inducing immune-mediated response and resulting in reduction of Aβ and tau load in AD animal models [6, 7, 14, 15, 33].

*Interfering with Aβ and tau production and aggregation: Aβ peptide inhibitor (SI), GSK-3 inhibitor (aminothiazole AR-A0144418) and protoporphyrin IX(PX).*

Liu et al. [8] reported that SI stabilized small soluble conformers of Aβ and saturated the early benefit of FUS-MB/BAM-10 treatment in TgCRND8 mice.GSK-3 served as the primary kinase responsible for Aβ peptide production by interfering with amyloid precursor protein (APP) cleavage at the α- and γ-secretase complex and tau phosphorylation modulated by insulin/insulin-like growth factor (IGF)-PI3K-Akt signaling pathway [51]. Hsu et al. [9] found that the GSK-3 inhibitor (AR-A014418) was delivered into the brain for GSK-3 downregulation to reduce Aβ peptide and phosphorylated tau in APPswe/PSEN1-De9 mice. Xu et al. [10] detected PX released into the brain from a nanocarrier by FUS which served as a substrate inhibitor of GSK3β, effectively reduce the phosphorylation of tau in APP/PS1 mice.

*Rescuing cholinergic function: TrkA agonist (D3).*

The cholinergic hypothesis of AD indicates that widespread neuronal and synaptic deficits, degeneration of basal forebrain cholinergic neurons (BFCNs), and loss of cholinergic innervation to the cortex (CTX) and hippocampal formation (HF) contribute to cognitive decline in AD[52]. Nerve growth factor (NGF) binding to TrkA triggers intracellular signaling via the mitogen-activated protein kinase (MAPK) and phosphoinositide 3-kinase (PI3K)/Akt cascades to promote neuronal survival, growth and synaptic plasticity in BFCNs[53, 54]. BFCNs respond to NGF-induced activation of TrkA, increasing ChAT activity and promoting Ach release in the HF and CTX[55]. Xhima et al. [13] demonstrated that TrkA agonist (D3) was delivered to basal forebrain using MRIgFUS activated TrkA dependent signaling cascades and enhanced cholinergic transmission in TgCRND8 mice.

*Production of reactive oxygen species (ROS): PX@OP@RVGs.*

The production of reactive oxygen species (ROS) is catalyzed by redox active metal ions bound to Aβ [56]. Xu et al. [10] proposed that the PX can induce the accumulation of ROS in the presence of FUS, contributing to the inhibitory effect on Aβ aggregation and toxicity in APP/PS1 mice.

*Suppression of endoplasmic reticulum (ER) stress: Qc@SNPs.*

Accumulated evidence shows that ER stress will cause oxidative damage inducing neuronal degeneration and neuroinflammation associated with the development of AD [57]. Liu et al. [12] illustrated that Qc released from its nanocarrier by FUS-MB could effectively reduce neuronal apoptosis, inflammatory response and the Aβ content caused by ER stress in APP/PS1 mice.

### FUS-MB treatment without therapeutic agents

FUS-MB treatment without therapeutic agents has been applied in mice, rats, rabbits, nonhuman primates, and even human in recent years. Repeated sonication is commonly used to enhance the therapeutic effect and appears not to cause short-term or long-term (4-20 months) side effects under proper exposure parameters [58-60]. In animal studies, Poon et al. also found that repeated FUS-MB treatment had additive effects in reducing Aβ plaque burden (number and surface area) in the targeted region. The sonication protocol with low central frequency (0.5 MHz and 0.715 MHz), low acoustic threshold (<0.7 MPa), 10ms pulse length, 1-10Hz PRF and total duration of 60s-120s was shown to open BBB without tissue damages. Real-time PCD were also applied to adjust the acoustic pressure within safe limits [16, 18].

The findings of included animal studies showed that a single or repeated FUS-MB treatment alone could reduce Aβ and tau burden, enhance cholinergic function, induce neurogenesis, and improve cognitive and memory deficits. The underlying mechanisms could include the following:

*FUS-MB induced BBB opening allowing the entry of endogenous antibodies.*

Three studies demonstrated that the entry of endogenous antibodies (IgG and IgM) binds to Aβ plaque, facilitating the opsonization and internalization by microglial and astrocyte [15, 16, 18].

*FUS-MB induced BBB opening allows the infiltration of systemic phagocytic immune cells into the brain.*

Immune cells can aid in Aβ and phosphorylated tau (p-tau) clearance [18, 19]. Karakatsani et al. [19]observed that immune cells could migrate to the contralateral-to-sonication hemisphere to reduce the whole brain p-tau burden.

*FUS-MB induced BBB opening activates astrocytes and microglia surrounding Aβ plaque.*

Activated astrocytes and microglia internalize Aβ and contribute to plaque reduction [15, 16, 34]. Leinenga et al. [17] detected that repeated SUS-MB broke down larger plaques into smaller pieces facilitating capture and degradation by activated microglia.

*FUS-MB-induced BBB opening increases cholinergic function and the expression of BDNF.*

FUS-MB treatment reduced acetylcholinesterase (AChE) activity, increased Ach release and promoted BDNF expression in the hippocampus, which upregulated neuroplasticity and neurogenesis (increased DCX+ and BrdU+ cells) via Akt signaling, resulting in improvements in cognitive and memory function [16, 21]. Shin et al. [21] found that FUS-MB treatment resulted in the recovery of Ach levels and promoted BDNF expression, contributing to the hippocampal neurogenesis in selective immunotoxin 192 IgG-saporin (SAP) rats. Two research groups provided evidence that repeated MRIgFUS treatment increased the proliferation and maturation of neuron cells in the targeted hippocampus in TgCRND8 mice [14, 16].

*FUS-MB induced BBB opening decreases the proinflammatory cytokine.*

Dubey et al. [14] showed that repeated MRIgFUS treatment reduced TNF-α in the hippocampus in TgCRND8 mice. TNF-α is known to inhibit neurogenesis and influence Aβ pathologies and cognitive deficits.

*FUS-MB treatment enhances the clearance of Aβ and tau through the ubiquitin pathway, autophagy pathway and glymphatic-lymphatic system.*

FUS induced BBB opening has previously been demonstrated to increase the ubiquitination of proteins specifically within neurons [61]. Nisbet et al. [7] proposed that their observation of the increased turnover of phosphorylated tau in pR5 mice happens through enhancement of the ubiquitin pathway induced by repeated SUS-MB treatment. However, Pandit et al. [20] detected no increase in ubiquitinated degradation of phosphorylated tau after repeated SUS-MB treatment. They found clearance of p-tau and NFTs via the autophagy pathway activated by repeated SUS-MB treatment in K3 mice [20]. The glymphatic system is a postulated waste system for cerebral spinal fluid (CSF)-interstitial fluid (ISF) exchange in the brain driven by the CSF influx force, which moves solutes from the periarterial CSF space via ISF efflux to the perivenous CSF space. Waste solutes (*i.e.* Aβ and tau) travel through the meningeal lymphatic system to the outside of the brain and are drained to deep cervical lymph nodes (dCLN) [62]. Lee, et al. [22] observed that repeated FUS-MB enhanced solute Aβ clearance from brain to the cerebrospinal fluid (CSF) space and deep cervical lymph nodes in 5XFAD mice, suggesting the beneficial effect of FUS-MB treatment upon Aβ removal through the glymphatic-lymphatic system. Memory improvement was also correlated with accumulation of Aβ in CSF. The authors speculated that MB cavitation in the arteries during sonication might function to mimic and enhance the arterial pulsatility, thus driving interstitial spinal fluid (ISF)-CSF efflux of Aβ solutes, contributing to the enhanced clearance of Aβ.

The application of FUS-MB treatment in AD patients remains under phase I and II clinical trials. These studies are focused on the feasibility, tolerability, and efficacy of repeated FUS-MB treatment. Transient and reversible BBB opening was seen in targeted regions (frontal lobe, entorhinal cortex and hippocampus) under sonication protocols using 220 kHz central frequency, 300 ms pulse length and 0.74% duty cycle for total 50s with 2-3 treatment sessions. Meng et al. [38] further detected enhanced distribution of gadolinium within the glymphatic pathway, including the perivascular space, SAS and space surrounding large veins draining toward the dural sinuses after FUS-MB treatment, suggesting glymphatic efflux persists after BBB opening in human. Most AD subjects tolerated the FUS procedure well and experienced no serious treatment-related adverse event (e.g., deaths, hemorrhage, swelling, short-term or long-term neurologic deficits). A few patients presented transient increases in neuropsychiatric assessment scores and headache. Meng, et al. [39] reported transient neural functional changes within the frontoparietal networks immediately after FUS-MB treatment that resolve within a day. Regarding the outcomes of FUS-MB treatment, the AD subjects showed no clinically meaningful improvement and [18F]-Florbetaben PET-CT scans exhibited no changes in Aβ deposition at 1 month and 3 months after FUS-MB treatment [40, 41]. However, the findings of safe BBB opening support the continued investigation of FUS as a potential novel treatment and drug delivery strategy for AD patients.

### FUS stimulation

Brain stimulation using FUS without MB has been developed to modulate neuronal activity without thermal effects. This FUS stimulation has aroused increasing interests as it holds the promise of a far better spatial resolution than other non-invasive stimulation techniques and the ability to reach deep brain areas [63].

There are a few FUS stimulation studies in AD animals or AD patients. In animal studies, higher frequencies (1-2 MHz) and longer sonication duration (5-60min) are applied. Spatial peak temporal average intensity (I_SPTA_) is related to the risk of thermal bio-effects and the spatial peak pulse average intensity (I_SPPA_) is associated with the risk of cavitation. These are two main indices for assessing safety. Two studies exploit low intensity pulsed ultrasound (LIPUS) with ranges of I_SPTA_ between 0.099 w/cm^2^ and 0.528 w/cm^2^ and no adverse effects (bleeds and neuronal loss) reported (28, 30). One study used higher I_SPTA_ (3.0 w/cm2) and I_SPTA_ (190 w/cm2) to target the hippocampus in 5XFAD mice [26]. Although this study did not mention treatment side effects, the applied of I_SPTA_ was below the international standard upper limit (IEC standard 60601-2-5) set for the “effective intensity”.

The animal studies in this review revealed that repeated FUS stimulation could induce neuronal plasticity and neurogenesis, increase cerebral blood flow (CBF), reduce Aβ plaque and microgliosis, and improve the cognitive function. The underlying mechanisms of FUS stimulation could include the following:

*Repeated LIPUS treatment attenuated AChE activity and enhanced the expression of neurotrophic factors.*

Two included studies showed that repeated LIPUS reduced AChE activity and increased the expression of brain-derived neurotrophic factor (BDNF), glial cell line-derived neurotrophic factor (GDNF) in hippocampus, which were associated with neurogenesis and the improvement of cognitive and memory function [23, 25].

*Repeated LIPUS treatment downregulated genes related to inflammation and expression of Aβ.*

Lin et al. found that repeated LIPUS stimulation attenuated Aβ1-42 expression [23]. Eguchi et al. detected that repeated LIPUS decreased the expression of amyloid precursor protein (APP) and β-site amyloid precursor protein cleaving enzyme-1 (BACE-1), resulting in reduction of Aβ plaque, along with reduced microgliosis [25].

*Repeated LIPUS upregulated the eNOS expression.*

Eguchi, et al observed that repeated LIPUS upregulated eNOS expression, which suppressed Aβ accumulation and associated glial cells activation and elevated CBF [25]. However, Bobola et al. found no meaningful production of eNOS after repeated FUS stimulation [26]. This may be due to the relatively higher intensity FUS used in this study having different effects on eNOS.

*Repeated FUS stimulation activated microglia.*

Bobola, et al. reported that FUS stimulation at 40Hz increase activated microglia colocalized with plaque and decreased Aβ load [26].

There are two clinical trials of FUS stimulation in AD patients. Nicodemus et al. used a focused transcranial Doppler device with 2MHz central frequency and low I_SPTA_ of 520mW/cm2. Beisteiner et al. used transcranial pulse stimulation based on ultrashort ultrasound pulses (PRF: 5Hz and pulse length: 3 μs). All participants tolerated FUS stimulation without side effects or clinical treatment related symptoms during FUS stimulation and up to the 3-month follow-up. Nicodemus et al. found FUS stimulation could improve cognitive and motor function [27]. Their ASL MRI scans also indicated that the incremental increased perfusion in the targeted regions after 1h FUS stimulation [27], which is consistent with the findings in animal studies and can be explained by upregulation of eNOS. Beisteiner et al. [28] observed that patients’ cognitive state was improved after FUS stimulation and remained stable over 3 months. They also confirmed that the increased activation in hippocampus and upregulation of memory network after FUS stimulation were correlated with cognitive performance, suggesting FUS stimulation has neuromodulation effects in humans. The possible underlying mechanisms of FUS neuromodulation include a) acoustic radiation forces effects on the permeability of ion channels, such as mechanosensitive channels and voltage-gated calcium, sodium and potassium channels. b) ultrasound generates nanobubbles in the lipophilic zone of the plasma membrane, which alters the local curvature of the bilayer and changes overall neuronal activities [64].

### Limitation and challenges

Numerous animal and postmortem human studies have confirmed BBB breakdown takes place in the AD brain, which exhibits extravascular leakage, pericyte and endothelial degeneration, as well as loss of BBB tight junctions [65, 66]. Recent imaging and biomarker studies showed an early BBB breakdown and vascular dysregulation in AD that is detectable before cognitive decline and/or other brain pathologies [67, 68]. Cerebral amyloid angiopathy (CAA) is regarded as the main cause of BBB disruption and one of the pathological hallmarks of AD [69, 70]. Despite the preliminary success of FUS-MB as a drug delivery method and stand-alone treatment for AD, several questions regarding the safety issue and the therapeutic effect of FUS in AD with CAA pathology and existing BBB disruption.

*Do safe parameters of FUS-MB for BBB opening differ across animal models?*

Studies included in this review showed the same FUS parameters for BBB permeability were applied on AD mice and wild-type controls, showing no significant difference in the mortality rate and no overt post-FUS side effects (such as hemorrhage) under the proper FUS parameters [9, 13-16, 18, 19, 22]. TgCRND8 mice have a double mutation of the amyloid precursor protein and are known to develop amyloid pathology by 2-3 months of age [15]. Only one study (Jordao et al. 2013) used twice the dose of MB (Definity) for 4 months old TgCRND8 mice, suggesting an altered vascular response to FUS in TgCRND8 mice [15]. Although several studies have demonstrated that the peak acoustic pressure induced subharmonic emission has no significant difference between TgCRND8 mice and non-Tg mice [13, 16, 71, 72], a recent study showed that TgCRND8 mice treated with vasculotide (neuroprotective properties of protection from BBB breakdown and reduction in neuroinflammation) can lower the threshold to sub- and ultra-harmonic bubble behavior [72], might be benefit for lowering the likelihood of adverse effects and death.

*Does CAA pathology affect BBB permeability and therapeutic effects after FUS-MB treatment?*

Our recruited studies demonstrated that there are no significant differences in the post-FUS BBB permeability of contrast medium, drugs (IVIg) and endogenous antibodies (IgG and IgM) between 4-7 months old TgCRND8 mice and non-Tg mice [13-16, 18]. However, Burgess et al recently observed the disparate leakage kinetics under similar acoustic pressures between TgCRND8 mice and non-Tg mice by using two-photon microscopy, exhibiting less fast leakage and increase slow leakage in TgCRND8 mice [73]. The mechanism of FUS-MB induced tracers or drugs cross the BBB has been proven to via widened tight junctions (paracellular) and transcytosis (transcellular) and the observation of fast and slow leakage kinetics has been postulated to corresponding to paracellular and transcellular transport [42, 44]. The findings of Burgess’ s study indicated that FUS does not exacerbate BBB dysfunction but promotes delivery of therapeutic molecules via the transcellular pathway. Regarding the discrepancy of therapeutic effects of FUS-MB treatment between TgCRND8 mice and non-Tg mice, Burgess et al. [16] detected no significant difference in the FUS-MB induced neurogenesis in the dentate gyrus, including immature neurons count and total dendrite path length, indicating that CAA pathology does not influence neurogenesis after FUS. Jordao et al. [15] found that elevation of GFAP levels (a marker of astrocytes) increased at 4 days after FUS-MB treatment and remained significantly high at 15 days in TgCRND8 mice, but not in non-Tg mice, suggesting that the CAA pathology or existing BBB opening exert additional effect of FUS-MB on the activation of astrocytes. But this study did not compare Aβ load (size, surface area) after FUS treatment between TgCRND8 mice and non-Tg mice. Whether CAA pathology has an impact on FUS-MB induced Aβ reduction remains unclear and needs further investigation.

*Does CAA pathology would affect the BBB restoration after FUS-MB treatment?*

It is known that tight junction proteins, including occludin, claudin-1, claudin-5 and ZO-1 play a key role in the “tightness” of endothelial tight junction and limit large molecules (>400Da) entering the brain [74]. A series of the specialized endothelial transporters, including solute carrier-mediated transporters, receptor-mediated transporters, ATP-binding cassette (ABC) transporters (e.g., P-glycoprotein (Pgp)) and ion transporters allow the exchanges of energy metabolites, nutrients, regulatory molecules and metabolic waste products [75]. FUS-MB has been demonstrated to temporarily reduce the expression of occludin, claudin-5, ZO-1 and Pgp. These tight junction proteins and Pgp were shown to be restored at 24 h and 72 h post-FUS in normal brains [74, 76] The restoration of the tight junction proteins and Pgp is regarded as the underlying mechanism of the reversibility of FUS induced BBB opening. Lynch et al. [72] showed that the BBB was impermeable to Evan’s Blue dye at 24h after FUS-MB treatment in both 5-7 months old TgCRND8 mice and non-Tg mice, suggesting that CAA pathology may not affect BBB closure. However, Evan’s Blue is a relatively large molecule (~70 kDa) that may produce more rapid closure time. This study did not investigate whether BBB was also impermeable to smaller molecules (such as gadolinium contrast agents of ~600 Da) within 24h post FUS in TgCRND8 mice. In addition, there is a lack of studies examining the changes of tight junction proteins and endothelial transporters after FUS-MB treatment in CAA or AD brains.

### Conclusion

FUS is a non-invasive technique that can be used for the treatment of AD. Current preclinical animal studies show effective drug delivery in the brain using FUS-MB and therapeutic results from FUS-MB treatment alone and FUS stimulation in AD models that correlate with cognitive improvement. In addition, early stages of clinical trials using FUS-MB treatment alone have also demonstrated FUS-MB can be safely administered to patients. FUS applied as a method for brain stimulation in patients has shown non-invasive increases in local blood flow and cognition in AD patients. However, device-related parameters still need further optimization to establish standardized and safe procedures for FUS in AD patients, who also have CAA pathology and BBB breakdown. Current clinical trials of FUS-MB treatment do not show a noticeable effect on reducing Aβ load and improving neurological symptom and there is also a lack of FUS-MB induced drug delivery attempts in AD patients. In the future, we expect to see increased understanding of FUS mechanism that should broaden the scope of clinical application of FUS.
